# Retinoic acid receptor γ activation promotes differentiation of human induced pluripotent stem cells into esophageal epithelium

**DOI:** 10.1007/s00535-020-01695-7

**Published:** 2020-06-16

**Authors:** Yasufumi Koterazawa, Michiyo Koyanagi-Aoi, Keiichiro Uehara, Yoshihiro Kakeji, Takashi Aoi

**Affiliations:** 1 Division of Advanced Medical Science, Graduate School of Science, Technology and Innovation, Kobe University, 7-5-1 Kusunoki-cho, Chuo-ku, Kobe, 650-0017 Japan; 2 Department of iPS Cell Applications, Graduate School of Medicine, Kobe University, Kobe, Japan; 3 Division of Gastrointestinal Surgery, Department of Surgery, Graduate School of Medicine, Kobe University, Kobe, Japan; 4 Center for Human Resource Development for Regenerative Medicine, Kobe University Hospital, Kobe, Japan; 5 Division of Pathology, Graduate School of Medicine, Kobe University, Kobe, Japan

**Keywords:** Induced pluripotent stem cells, Esophageal epithelial cell differentiation, Retinoic acid signal, Retinoic acid receptor γ

## Abstract

**Background:**

The esophagus is known to be derived from the foregut. However, the mechanisms regulating this process remain unclear. In particular, the details of the human esophagus itself have been poorly researched. In this decade, studies using human induced pluripotent stem cells (hiPSCs) have proven powerful tools for clarifying the developmental biology of various human organs. Several studies using hiPSCs have demonstrated that retinoic acid (RA) signaling promotes the differentiation of foregut into tissues such as lung and pancreas. However, the effect of RA signaling on the differentiation of foregut into esophagus remains unclear.

**Methods:**

We established a novel stepwise protocol with transwell culture and an air–liquid interface system for esophageal epithelial cell (EEC) differentiation from hiPSCs. We then evaluated the effect of all-trans retinoic acid (ATRA), which is a retinoic acid receptor (RAR)α, RARβ and RARγ agonist, on the differentiation from the hiPSC-derived foregut. Finally, to identify which RAR subtype was involved in the differentiation, we used synthetic agonists and antagonists of RARα and RARγ, which are known to be expressed in esophagus.

**Results:**

We successfully generated stratified layers of cells expressing EEC marker genes that were positive for lugol staining. The enhancing effect of ATRA on EEC differentiation was clearly demonstrated with quantitative reverse transcription polymerase chain reaction, immunohistology, lugol-staining and RNA sequencing analyses. RARγ agonist and antagonist enhanced and suppressed EEC differentiation, respectively. RARα agonist had no effect on the differentiation.

**Conclusion:**

We revealed that RARγ activation promotes the differentiation of hiPSCs-derived foregut into EECs.

**Electronic supplementary material:**

The online version of this article (10.1007/s00535-020-01695-7) contains supplementary material, which is available to authorized users.

## Introduction

The esophagus is known to be derived from the anterior region of the foregut via tracheal–esophageal separation in embryogenesis. Previous studies using mouse models have identified several important signals in the development of esophageal epithelium [1, 2]. However, the molecular mechanisms regulating this process remain to be fully clarified. In particular, relatively little information is available concerning the developmental process of human esophageal epithelial cells (EECs).

Human tissue organoids derived from human pluripotent stem cells have proven powerful tools for research on the developmental biology of various human organs [3]. For example, several reports using *human-induced pluripotent stem cells (hiPSCs) have identified signals and transcriptional factors that play essential roles in the development of foregut-derived organs, such as the liver, lung and stomach [4–6]. However, there have been only two reports concerning the generation of human esophagus from iPSCs [7, 8], and neither resolved molecular mechanisms underlying the differentiation protocols employed.

All-trans retinoic acid, which binds to RARα, RARβ and RARγ as a pan RAR agonist, has shown to promote differentiation of various endodermal tissues, including lung, pancreas and bladder [5, 9, 10], and tissues consisting of squamous cells, such as skin and cornea [11, 12], in several studies using human cells. In mouse models, retinoid acid signaling enhanced the terminal differentiation and proliferation of esophageal progenitor cells [13, 14], but the effects of retinoid acid signaling on the earlier developmental processes of esophagus have never been reported. Regarding the process of differentiation from human foregut into esophageal epithelium, the effect of retinoic acid signaling remains unclear.

In this study, we generated human esophageal epithelium from hiPSCs using a novel stepwise differentiation protocol and evaluated the effect of retinoic acid signaling on the processes. We found that RARγ activation promotes the differentiation of human anterior foregut into EECs.

## Methods

### iPSC culture

We used the iPSC line FF-PB-3AB4, which was established from a healthy donor’s peripheral blood mononuclear cells (PBMCs) [15]. The institutional review board of the Kobe University Graduate School of Medicine approved this study (No. 1722), and informed consent was obtained from the donor.

We cultured the iPSC line FF-PB-3AB4 according to a previously described method [16]. In brief, the iPSCs were cultured with iMatrix-511 silk (0.25 μg/cm^2^; Nippi, Tokyo, Japan) in uncoated usage and maintained in StemFit medium (Ajinomoto, Tokyo, Japan) with 50 U/ml of penicillin and 50 μg/ml of streptomycin (Life Technologies, Waltham, MA, USA) at 37 °C with 5% CO_2_. The medium was changed every other day. The cells were passaged every 5–8 days using 0.5 × TrypLE Select (1 × TrypLE Select diluted 1:1 with 0.5 mM EDTA/PBS [–]; Life Technologies) and cultured in StemFit medium supplemented with 10 μM of Rock inhibitor (Y-27632; WAKO, Osaka, Japan) for 1 day.

### EEC differentiation

For differentiation into definitive endoderm (DE), 1.8 × 10^5^ iPSCs were seeded as single cells on 24-well plates pre-coated with iMatrix-511 silk (0.5 μg/cm^2^) in StemFit medium supplemented with 10 µM of ROCK inhibitor Y-27632 (WAKO). The next day, the medium was changed to RPMI 1640 medium (Nacalai Tesque, Kyoto, Japan) supplemented with 100 ng/ml of Activin A (Peprotech, Rocky Hill, NJ, USA), 1 or 3 µM of CHIR99021 (TOCRIS, Bristol, UK), 2% B27 (Life Technologies), 2 mM *L*-glutamine (Life Technologies), penicillin and streptomycin, and cells were cultured until Day 3.

To generate foregut (FG), cells were cultured with RPMI 1640 medium supplemented with 2% fetal bovine serum (FBS), 2 µM of CHIR99021 (TOCRIS), 100 ng/ml of LDN193189 (Cellagen Technology, San Diego, CA, USA), penicillin and streptomycin from Day 3 to Day 6. SB431542 (5 μM; WAKO) was added on Day 4 and Day 5.

For induction of dorsal anterior foregut (dAFG), cells were cultured with RPMI 1640 medium supplemented with 2% FBS, 2 µM of CHIR99021 (TOCRIS), 0.5 µM ATRA (WAKO), 10 ng/ml of FGF10 (WAKO), 10 ng/ml of KGF (WAKO), 10 ng/ml of BMP4 (R&D Systems, Minneapolis, MN, USA), 10 ng/ml of EGF (R&D Systems), penicillin and streptomycin from Day 6 to Day 13.

**Fig. 1:**
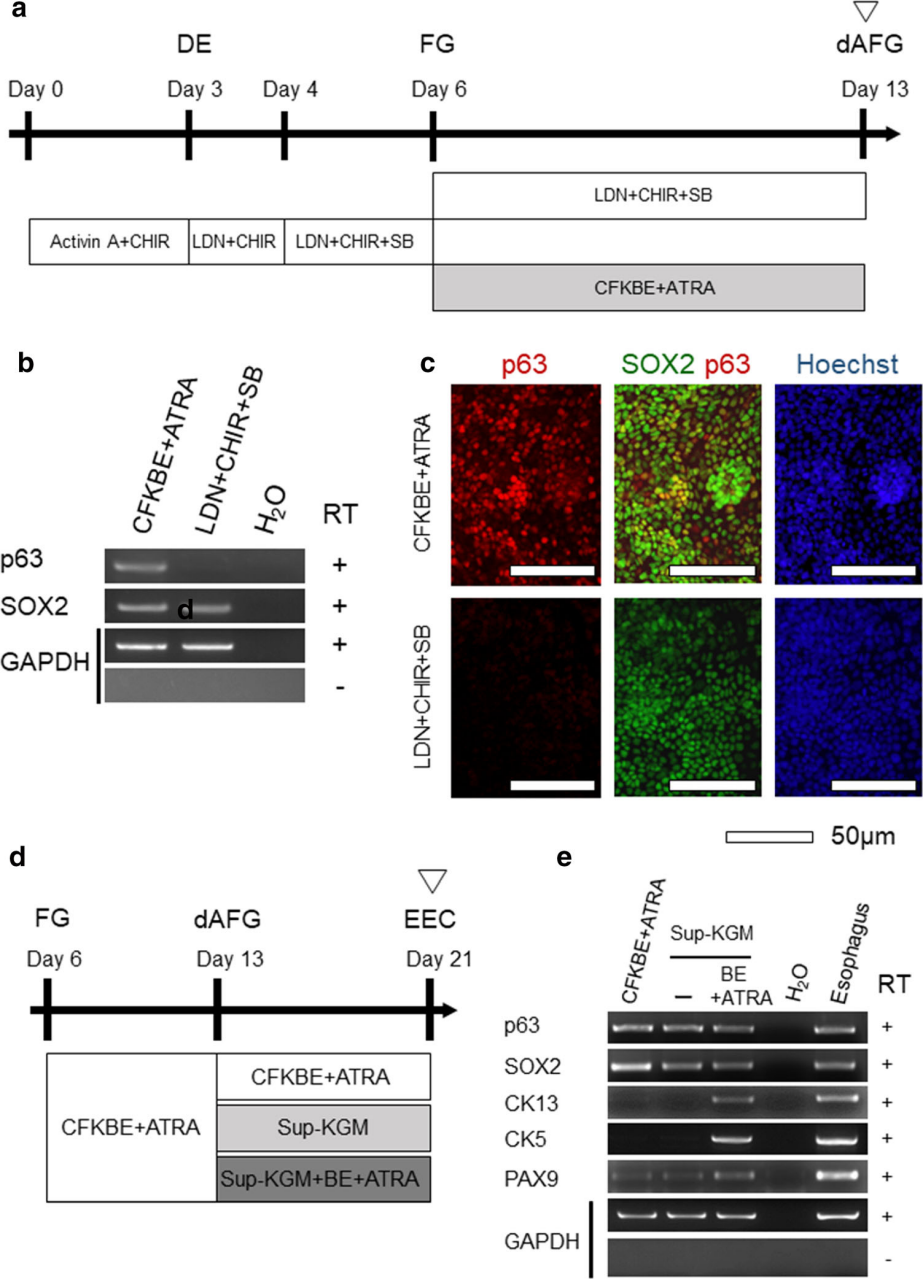
Induction of EECs from hiPSCs. **a** A schematic diagram of the experiment to examine the conditions underlying the differentiation of foregut (FG) into dorsal anterior foregut (dAFG). **b** An expression analysis of SOX2 and p63 at Day 13 by semi-quantitative RT-PCR. GAPDH was used as an endogenous control. **c** Immunostaining of SOX2 (green), p63 (red) and Hoechst (blue) at Day 13. Representative fluorescence is shown. Scale bars, 50 μm. **d** A schematic diagram of the experiment to examine the conditions underlying the differentiation of dAFG into EECs. **e** Expression analyses of p63, SOX2, CK13, CK5 and PAX9 at Day 21 by semi-quantitative RT-PCR. GAPDH was used as an endogenous control. Total RNA of human normal esophageal tissue was used as a positive control

**Fig. 2:**
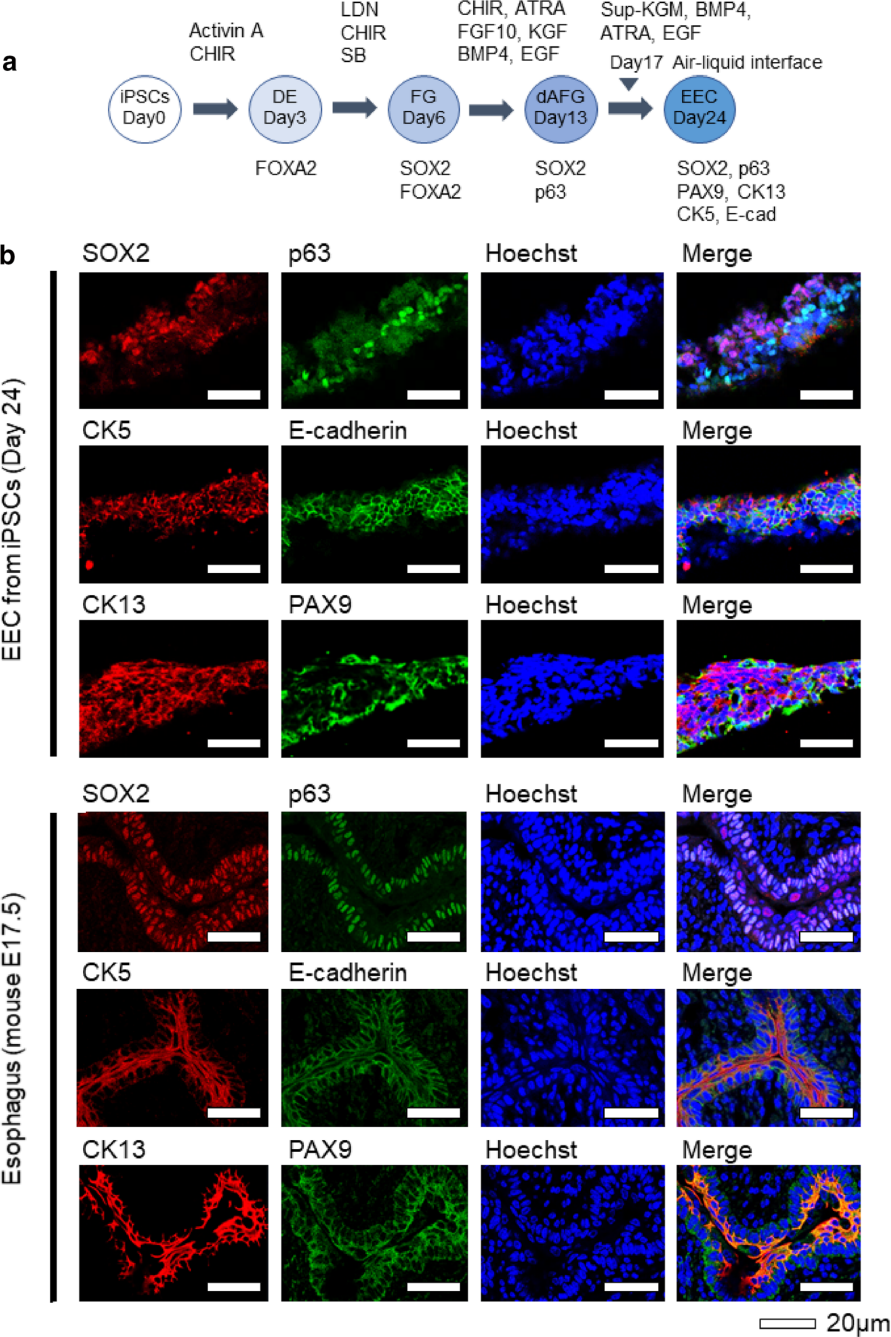
The expression of esophageal epithelial marker proteins in the hiPSC-derived cells and fetal mouse esophagus at E17.5. **a** A schematic representation of the stepwise differentiation protocol and marker genes at each step. **b** Immunostaining of the SOX2, p63, CK5, E-cadherin, CK13 and PAX9 at Day 24 in the derivatives of hiPSCs (upper panels) and fetal mouse esophagus at E17.5 (lower panels). Scale bars, 20 μm

**Fig. 3:**
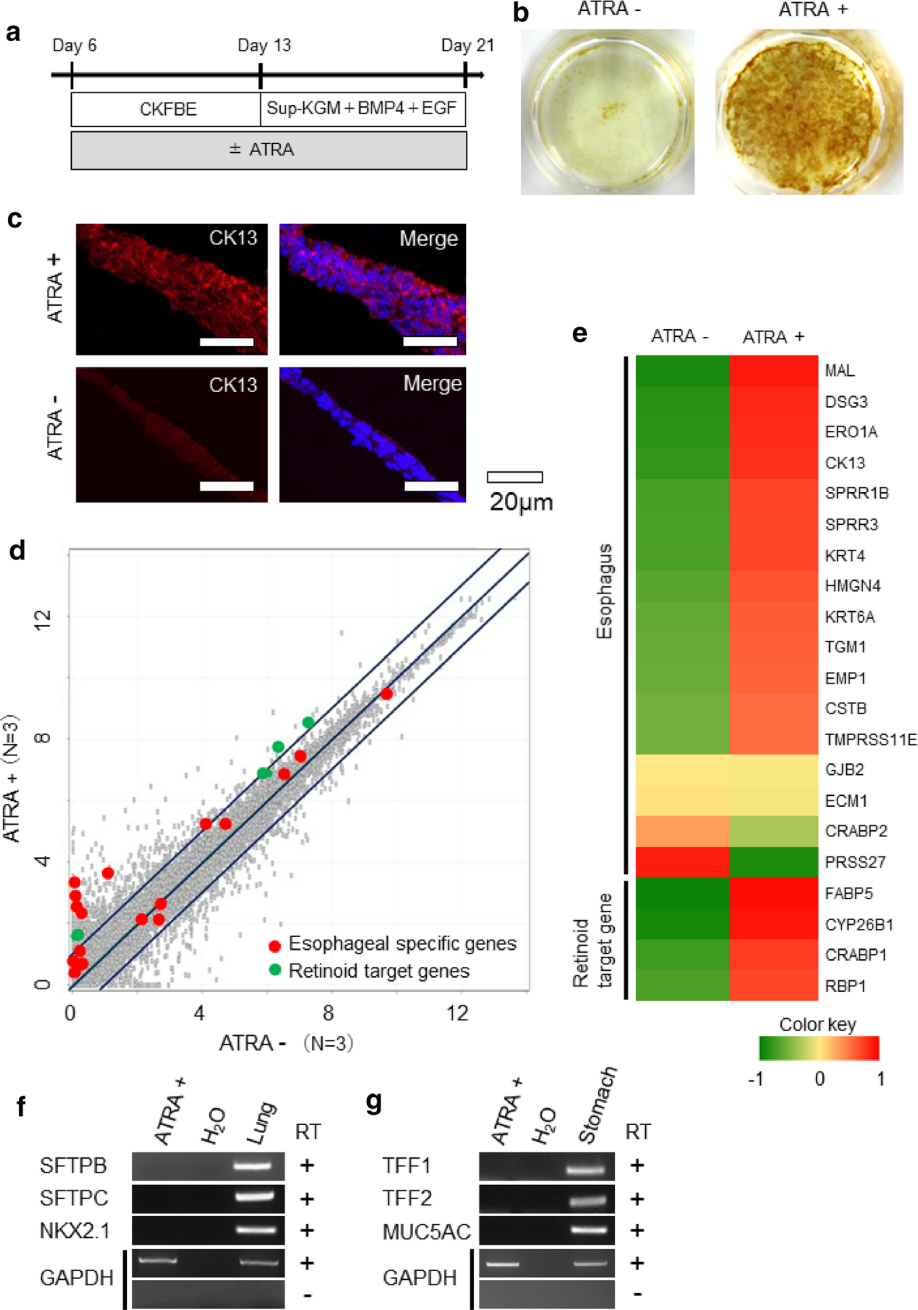
Enhancing effects of ATRA on the esophageal epithelial differentiation. **a** A schematic diagram of the experiment to examine the effects of ATRA on the differentiation of foregut cells into esophageal epithelium. **b** Lugol staining at Day 21. **c** Immunostaining of CK13 (red) at Day 21 (left panels). The right panels show merged images of CK13 and Hoechst (blue) staining. Scale bars, 20 μm. (**d**, **e**) Transcriptome data analyses of the cells derived with or without ATRA at Day 21. The scatterplot shows the expression of esophageal-specific genes (red dots), retinoid target genes (green dots) and other genes (gray dots) (**d**). The heat map shows the average Z-scores of esophageal-specific genes and retinoid target genes (**e**). **f** RT-PCR analyses of the lung marker genes SFTPB, SFTPC and NKX 2.1 at Day 21. GAPDH was used as an endogenous control. Total RNA of human normal lung tissue was used as a positive control. (**g**) RT-PCR analyses of stomach marker genes: TFF1, TFF2 and MUC5AC. GAPDH was used as an endogenous control at Day 21. Total RNA of human normal stomach tissue was used as a positive control

**Fig. 4:**
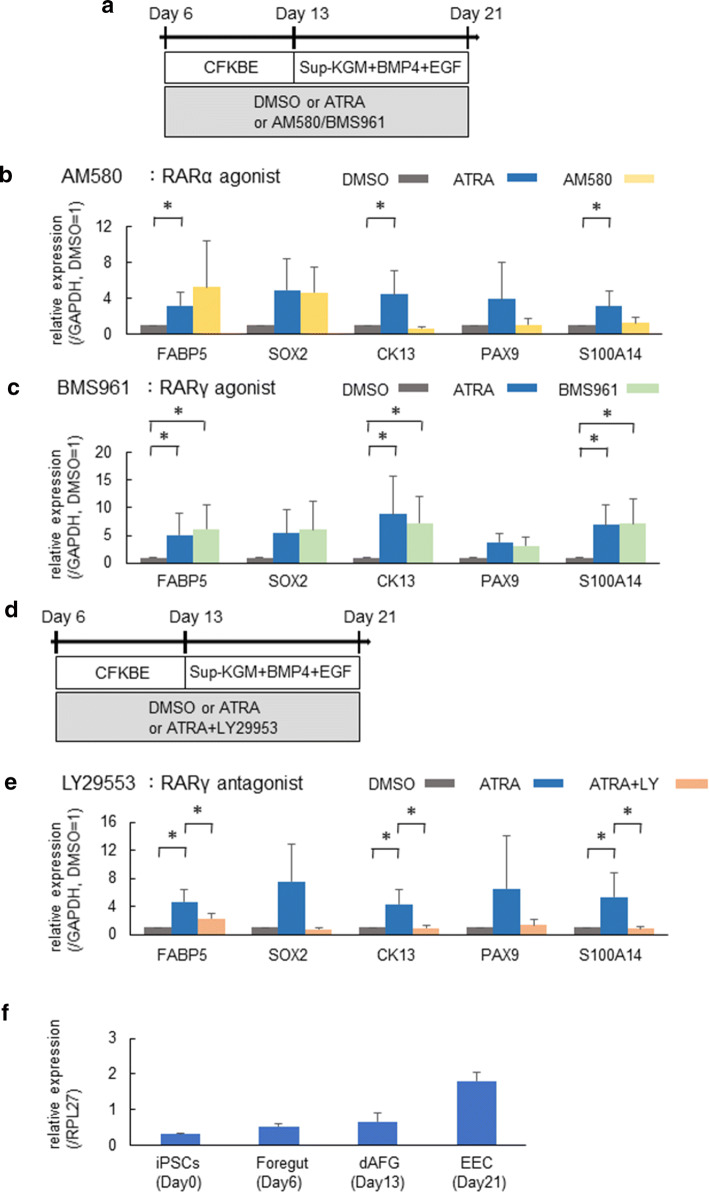
ATRA enhances differentiation into EECs via RARγ receptor. **a** A schematic diagram of the experiment to compare the effects of AM580 (RARα-specific agonist), BMS961 (RARγ-specific agonist) and ATRA on EEC differentiation. (**b**, **c**) Expression analyses of FABP5, SOX2, CK13, PAX9 and S100A14 in the differentiated cells treated with AM580 (**b**) or BMS961 (**c**) by qRT-PCR. GAPDH was used as an endogenous control. Data represent the mean ± SEM (*n* = 6). *p < 0.05 from an ANOVA with Tukey’s test. **d** A schematic diagram of the experiment to examine the effects of adding LY2955303 (RARγ antagonist) to ATRA treatment on EEC differentiation. **e** Expression analyses of FABP5, SOX2, CK13, PAX9 and S100A14 in differentiated cells treated with LY29953 by qRT-PCR. GAPDH was used as an endogenous control. Data represent the mean ± SEM (*n* = 6). *p < 0.05 from an ANOVA with Tukey’s test. (f) Expression analyses of RARγ in iPSCs (Day 0), FG (Day 6), dAFG (Day 13) and EECs (Day 21). RPL27 was used as an endogenous control. Data represent the mean ± SEM (*n* = 3)

**Fig. 5:**
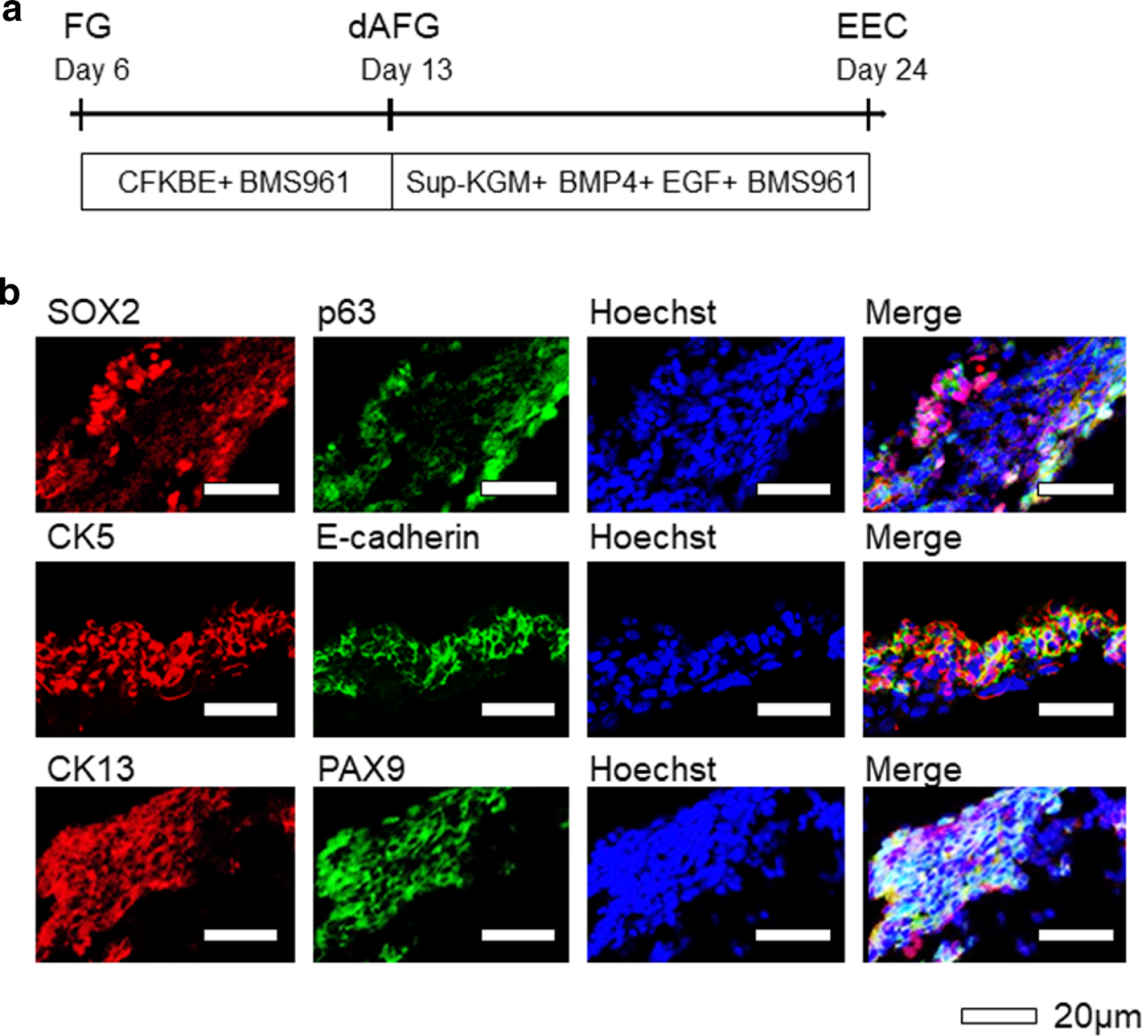
Treatment with an RARγ-specific agonist instead of ATRA also induced stratified layers of cells expressing esophageal epithelial markers. **a** A schematic diagram of the EEC differentiation protocol with an RARγ-specific agonist. **b** Immunostaining of SOX2, p63, CK5, *E*-cadherin, CK13 and PAX9 in the derivatives of hiPSCs. Scale bars, 20 μm

For terminal differentiation into EECs, cells were cultured with RPMI 1640 medium supplemented with KGM-Gold SingleQuots (Sup-KGM), containing 0.1% hydrocortisone, 0.1% transferrin, 0.05% epinephrine 0.25 ml/L, 0.1% GA-1000, 0.4% bovine pituitary extract, 0.1% human epidermal growth factor, insulin 0.5 ml/L (LONZA, Basel, Switzerland), 10 ng/ml of BMP4, 0.5 µM ATRA, 10 ng/ml of EGF, penicillin and streptomycin from Day 13 to Day 21 (Figs. 1, 3, 4) or Day 24 (Figs. 2, 5). Sup-KGM is a supplement for keratinocyte basal medium (Lonza) and used for differentiation into bladder urothelial cells, which also have stratified structures [15].

### RA receptor-specific agonists and antagonists

To assesses which RA receptor subtype plays an essential role in esophageal differentiation, 1 µM of the RARα agonist AM580 (Cayman Chemical, Ann Arbor, MI, USD), 2 µM of the RARβ agonist CD2314 (R&D Systems) and 0.5 µM of the RARγ agonist BMS961 (R&D Systems) were added from Day 6 to Day 21 (Fig. 4) or Day 24 (Fig. 5). In addition, 0.5 µM of ATRA with 2 µM of the RARγ antagonist MM11253 (R&D Systems) or 4 µM of the RARγ antagonist LY2955303 (R&D Systems) were added from Day 6 to Day 21.

### Transwell culture with air–liquid interface (ALI)

For the in vitro 3D reconstruction, 1.8 × 10^5^ iPSCs were seeded onto transwells (0.4-μm pore size; Corning, Corning, NY, USA) pre-coated with iMatrix-511 silk (0.5 μg/cm^2^). The protocol of differentiation was the same as that of normal culture. The ALI procedure was started on Day 17, and cells were exposed to the ALI by removal of the medium in the upper compartment until Day 24 [17].

### Semi-quantitative or real-time quantitative reverse transcription polymerase chain reaction (RT-PCR)

Total RNA was isolated using Trizol (Life Technologies) and treated with the TURBO DNA-free kit (Thermo Fisher Scientific, Waltham, MA, USA). The Prime Script II 1st strand cDNA Synthesis Kit (Takara, Shiga, Japan) was used to synthesize cDNA from 500 to 800 ng of total RNA.

For semi-quantitative RT-PCR (Figs. 1b, e, 3f, g, S-Figs. 2a, 2b, 8a, 8b), the resulting cDNA was subjected to PCR with a Takara Ex Taq PCR kit (Takara). In Figs. 4b, c, e, f and S-Figs 1d, 4b, 6b, 6d, 7b, 7c, a real-time quantitative RT-PCR analysis was performed using TB Green Premix Ex Taq (Takara) on a Light Cycler 480 II (Roche, Basel, Switzerland). The PCR primers used are listed in Supplementary Table 1.

### Immunocytochemistry

Cells were fixed with 10% formalin for 10 min at room temperature. After being washed with PBS, the cells were treated with 0.2% Triton X-100 in PBS and blocked with 5% donkey serum and 1% BSA in PBS for 45 min at room temperature. The cells were incubated with primary antibodies at 4 °C overnight and then stained with secondary antibodies. The primary antibodies were rabbit anti-SOX2 (ab97959, dilution 1:200; abcam, Cambridge, UK) and goat anti-p63 (BAF1916, dilution 1:200; R&D Systems). All of the secondary antibodies (Alexa Fluor 488-conjugated anti-rabbit IgG and Alexa Fluor 594-conjugated anti-goat IgG) were obtained from Life Technologies. Hoechst 33,342 (WAKO) was used for nuclear staining.

### Frozen section samples

The cultured organoids were fixed with 10% formalin and then embedded in Tissue-Tek O.C.T Compound (Sakura Finetek Japan, Tokyo, Japan) and frozen at − 80 °C. The frozen samples were sectioned at 8 µm on a cryostat.

### Immunohistochemical analyses of the organoids

For immunohistofluorescence, the sections were treated with 0.2% Triton X-100 in PBS and blocked with 5% donkey serum and 1% BSA in PBS for 45 min at room temperature. Primary antibodies were incubated overnight at 4 °C. Slides were washed in PBS and incubated with secondary antibody for 1 h at room temperature. The primary antibodies were rabbit anti-SOX2 (ab97959; abcam), rabbit anti-CK13 (ab92551, dilution 1:200; abcam), goat anti-p63 (BAF1916, dilution 1:200; R&D Systems), rat anti-PAX9 (ab28538, dilution 1:200; abcam), goat anti-*E*-cadherin (AF648, dilution 1:200; R&D Systems), rabbit anti-CK5 (GTX113219, dilution 1:200; GeneTex, Irvine, CA, USA), rabbit anti-Claudin4 (ab210796, dilution 1:200; abcam) and rabbit anti-Involucrin (ab53112, dilution 1:200; abcam). All of the secondary antibodies (Alexa Fluor 594-conjugated anti-mouse or anti-goat IgG, Alexa Fluor 488-conjugated anti-mouse, anti-goat, anti-rat or anti-rabbit IgG) were obtained from Life Technologies. Hoechst 33342 (WAKO) was used for nuclear staining.

### Lugol staining

After being washed with PBS, the cells were treated with lugol solution (1.2% potassium iodine and 0.6% iodine in water) and then washed again with PBS. Lugol solution was kindly provided by Kobe University Hospital (Kobe, Japan).

### RNA sequencing

Total RNA was isolated using Trizol (Life Technologies) and treated with the TURBO DNA-free kit (Thermo Fisher Scientific), as described above. The RNA was sent to Macrogen (Seoul, South Korea, https://www.macrogen.com) for library preparation and paired-end RNA sequencing on the Illumina Novaseq6000 platform. Raw sequence files (fastq) were aligned to the human transcriptome (hg38) reference sequences using the Strand NGS software program (Strand Life Science, Karnataka, India) with default parameters. The aligned reads were normalized using Transcripts per million (TPM) again with the Strand NGS software program. RNA-seq data have been deposited in the Gene Expression Omnibus (GEO) under accession number GSE140767.

### Extraction of esophagus-specific genes and Z score transformation

We defined tissue-specific markers as those with a > fivefold higher average Fragments per kilobase of exon per Million mapped fragments (FPKM) level in esophagus tissue than in all other tissues [18]. After normalizing with TPM, we performed log base two transformation. The average Z-scores of tissue-specific genes were then calculated using Excel 2016 (Microsoft, Redmond, WA, USA), and a heat map was created using Excel 2016 (Microsoft).

### Statistical analyses

All data were analyzed using the JMP^®^ 10 software program (SAS Institute Inc., Cary, NC, USA). Differences in the mean values between two groups were analyzed using the paired *t* test, and three groups were analyzed using an analysis of variance Tukey test. The differences were considered to be statistically significant for *P* values < 0.05 (*).

## Results

### Induction of EECs from hiPSCs

Based on previously reported FG endoderm induction protocols [4–6, 10, 19–22], we developed an original stepwise differentiation method from hiPSCs into EECs via DE, FG and dAFG.

First, hiPSCs were induced to differentiate into DE by treatment with the WNT activator CHIR99021 (hereafter CHIR) and Activin A for 3 days. We then differentiated DE into FG with a combination of LDN193189 (hereafter LDN) and SB431542 with CHIR. We confirmed the obvious expression of the FG marker genes SOX2 and FOXA2 by RT-PCR and immunocytochemistry (S-Fig. 1a and b). Since previous reports have shown that efficient FG induction requires an appropriate concentration of CHIR during DE induction [15, 19], 

we examined the expression of the FG marker SOX2 and mid/hindgut marker CDX2 at Day 6 when cultured with 1 or 3 μM of CHIR during DE induction (S-Fig. 1c) [23]. Quantitative PCR showed that 1 μM CHIR treatment induced a significantly higher expression of SOX2 and lower expression of CDX2 than 3 μM CHIR treatment (S-Fig. 1d). Based on these results, we used 1 μM of CHIR for DE induction in our protocol.

Next, to induce differentiation of FG into dAFG, we tested two conditions: (1) LDN, CHIR and SB431542 (LDN + CHIR + SB) or (2) CHIR, FGF10, KGF, BMP4, EGF and ATRA(CFKBE + ATRA) [20] (Fig. 1a). At Day 13, (2) resulted in an enhanced expression of p63, which is a marker for dAFG (esophageal progenitor) and is expressed in basal cells of the esophageal epithelium [2, 24], at the mRNA level (Fig. 1b) and protein level (Fig. 1c). In contrast, NKX2.1, a marker for ventral AFG (lung progenitor) and GATA4, a marker for posterior FG (gastric progenitor), were not expressed at this point (S-Fig. 2a and 2b, respectively).

To induce differentiation of dAFG cells into esophageal lineages, we tested three conditions after Day 13 (Fig. 1d). The first condition involved continued culture under the same conditions as before Day 13 (CFKBE + ATRA). Under the second condition, we used Sup-KGM (see the “Materials and Methods “section), as we previously succeeded in generating bladder urothelium, which shares stratified epithelial structures with esophageal epithelium, using Sup-KGM [15]. Under the third condition, we omitted lung specification reagents (CHIR, FGF10 and KGF) [20] from the CFKBE + ATRA cocktail and added Sup-KGM. After 8 days’ culture, the expression of esophagus marker genes (SOX2, p63 and PAX9) were detected under all three conditions. However, the expression of the mature esophageal epithelial markers CK5 and CK13 [21] was detected only in the cells generated under the third condition (Sup-KGM + BE + ATRA) (Fig. 1e).

### Expression of esophageal marker genes in the differentiated cells with ALI culture

A stratified structure is an important property of esophageal epithelium. We, therefore, employed ALI culture on a membrane (transwell culture system) as ALI culture enable us to assess vertical sections of the epithelium [25, 26] and has been reported to induce stratified structure formation of primary EEC culture [27]. Our differentiation protocol and the marker genes at each step are shown in Fig. 2a. On Day 24, we performed immunohistochemistry for the derivatives. We deduced that, like derivatives of hiPSCs in previous studies [7, 8], the derivatives in the present study would exhibit fetal histology but not adult histology, which would probably require more long-term differentiation culture. We,, therefore, used esophagus tissues of fetal mice (E17.5) as positive controls, as we were unable to obtain human fetal esophagus, which was the preferable positive control. Mouse esophagus is known to have keratinized layers, while human esophageal epithelium is not keratinized, and cells retain their nucleus [21]. Our HE staining findings confirmed this species difference in keratinization (S-Fig.3).

All of the esophageal marker genes we tested (SOX2, p63, CK5, E-cadherin, CK13, PAX9, Claudin-4 and Involucrin) were obviously expressed in the differentiated cells from hiPSCs (Fig. 2b and S-Fig. 3, upper panels), similar to the esophagus of mice (E17.5) (Fig. 2b and S-Fig. 3, lower panels). The findings of HE staining (S-Fig. 3) and the obvious expression of cell–cell adhesion molecules (*E*-cadherin and Claudin-4) suggested that the cells differentiated with our protocol formed stratified EEC layers but not piled-up collective cells without cell–cell adhesion. Notably, CK13 is expressed in the suprabasal and apical region but not in the basal region in authentic esophageal epithelium [8, 21]; all layers were positive for CK13 in our hiPSC-derived tissues, indicating that the tissues did not identically recapitulate an authentic human esophageal epithelium structure.

### Enhancing effects of ATRA on esophageal epithelial differentiation

We next evaluated the effects of ATRA in our esophageal differentiation protocol (Fig. 3a), as the effects of ATRA in the differentiation of FG have not been clarified [8, 10, 20]. First, we differentiated the cells into an esophageal lineage with 0, 0.1, 0.5 and 1.0 μM of ATRA from Day 6 (FG) to Day 21 (EEC) (S-Fig. 4a) to identify the appropriate concentration of ATRA for this study. Quantitative PCR showed that 0.5 and 1.0 μM ATRA treatment induced a higher expression of SOX2, CK13 and FABP5 than other concentrations (S-Fig. 4b). Considering the toxicity, the lower concentration of 0.5 µM was used for the subsequent experiments. The differentiation protocol devoid of ATRA obviously resulted in reduced staining of glycogen with lugol staining (Fig. 3b) and CK13 according to immunohistology (Fig. 3c), suggesting that ATRA plays a critical role in differentiation into esophagus.

We then analyzed the transcriptome data of the cells derived with or without ATRA. The scatterplot (Fig. 3d) and Z-score heatmap (Fig. 3e) showed the upregulation of 13 of the 17 esophagus-specific genes that were extracted from publicly available RNA-seq data (see the “Material and Methods” section) as well as known retinoid target genes [28] in the ATRA( +) cells.

Furthermore, since RA signaling is reported to have positive effects on lung [20] and posterior FG differentiation [8], we examined the expression of marker genes of lung (SFTEB, SFTPC and NKX2.1) and stomach, which is the posterior FG derivative, (TFF1, TFF2 and MUC5AC) in ATRA( +) differentiated cells using semi-quantitative RT-PCR (Fig. 3f, g). All of these marker genes were not expressed in the differentiated cells.

These data indicated that ATRA had a positive effect on the process of differentiation into esophageal epithelium.

### Effects of ATRA on differentiation from FG to dAFG and from dAFG to EECs

The effects of ATRA on organ specification from FG have been controversial [8, 20] (S-Fig. 5; also, see the “discussion” section). To clarify whether or not ATRA promotes differentiation of FG into dAFG in our protocol, we compared the expression of the dAFG marker genes SOX2 and p63 on Day 13 in the presence and absence of ATRA using quantitative RT-PCR (S-Fig. 6a). Our findings showed that ATRA significantly enhanced the expression of these marker genes as well as FABP5, a known target gene of ATRA (S-Fig. 6b).

We next assessed the effects of ATRA on the differentiation between dAFG and EEC (S-Fig. 6c), as no previous report has clarified the effect of ATRA treatment at this step (S-Fig. 5b, 5c). The results showed that, during transition from dAFG to EEC as well as from FG to dAFG, ATRA enhanced the expression of the EEC marker genes FABP5 and CK13 at Day 24 in the protocol (S-Fig. 6d).

### Effects of ATRA on differentiation into EECs via RARγ

ATRA upregulates the downstream genes through RAR and RXR heterodimers. Of the three subtypes of RARs, RARα and RARβ are widely expressed in many tissues, while the expression of RARγ is limited but particularly strong in the skin and esophagus (S-Fig. 7a) [18].

To identify which subtype of RARs is important for esophageal differentiation, we used the small molecules AM580 (RARα-specific agonist), CD2314 (RARβ-specific agonist) and BMS961 (RARγ-specific agonist) instead of ATRA (Fig. 4a and S-Fig. 7b) from Day 6 (FG) to Day 21 (EEC). RT-PCR showed that AM580 and CD2314 treatment resulted in a non-significant increasing trend in the expression of FABP5, which is a retinoid target gene [28] and whose expression is highest in the esophagus among human organ tissues [18], and SOX2, a general FG marker, but not the EEC-specific markers CK13, PAX9 and S100A14 (Fig. 4b and S-Fig. 7b). In contrast, BMS961 treatment significantly enhanced the expression of FABP5, CK13 and S100A14, similarly to ATRA treatment (Fig. 4c).

Therefore, in order to confirm whether or not ATRA enhances the differentiation of human esophagus via RARγ, we compared the expression of the esophageal markers in the resultant cells in the presence or absence of the RARγ antagonist LY2955303 during differentiation culture with ATRA (Fig. 4d). As shown in the Fig. 4e, RARγ antagonist treatment cancelled the enhancing effects of ATRA on the expression of esophageal-related genes, such as FABP5, SOX2, CK13, PAX9 and S100A14. Furthermore, to exclude the possibility that some off-target effect of LY2955303 contributed to the results of the experiments, we also used another RARγ antagonist, MM11253, and obtained the same results (S-Fig. 7c). In addition, we found that RARγ was expressed in the FG (Day 6), and the expression increased as the differentiation progressed (Fig. 4f). These results indicate that the enhancing effect of ATRA on esophageal differentiation depends on RARγ activation.

In addition, to address whether or not the increased SOX2 expression induced by RARα and RARβ agonist (Fig. 4b and S-Fig. 7b) meant that these agonists induced differentiation into other tissues expressing SOX2, such as stomach and neuronal cells [18], we examined the expression of marker genes of stomach (TFF1, TFF2 and MUC5AC) and neuronal cells (SOX1 and PAX6) in the cells treated with RARα and RARβ agonist by semi-quantitative RT-PCR. None of these marker genes was expressed in the cells (S-Fig. 8a and b), indicating that neither RARα nor RARβ agonist induced differentiation into these kinds of cells.

### Treatment with an RARγ-specific agonist instead of ATRA to induce stratified layers of cells expressing esophageal epithelial markers

Finally, we examined whether or not treatment with an RARγ agonist instead of ATRA could also induce cells that recapitulated the expression of esophageal differentiation marker proteins and stratified squamous epithelial structures (Fig. 5a). After culture with the RARγ-specific agonist using ALI, immunohistological analyses showed stratified cells with the obvious expression of SOX2, p63, CK5, E-cadherin, CK13, PAX9, Claudin-4 and Involucrin (Fig. 5b and S-Fig. 9).

## Discussion

We successfully generated stratified cells expressing esophageal epithelial markers from hiPSCs with our original method and evaluated the significance of RA signaling on EEC induction after FG differentiation. RA signaling is known to be involved in the development of various tissues, such as brain, limbs, eye, lung and pancreas [29, 30]. However, the effect of RA signaling on the esophageal development remains to be clarified.

Several previous studies using mouse models [13, 14], but none using human cells, have reported that ATRA enhanced the terminal differentiation and proliferation of esophageal progenitor cells. However, these mouse studies did not address the role of RA signaling in the early steps of esophageal development. There have been only two previous papers on hiPSCs-derived esophageal differentiation. One of them did not use any RA signaling agonists at all in their protocol. The other (Triso et al. [8]) evaluated the effect of ATRA before (but not after) FG differentiation. The present study demonstrated that ATRA promotes both progression from FG to dAFG, which is an esophageal progenitor (S-Fig. 6b), and from dAFG to esophageal epithelial differentiation (S-Fig. 6d) using hiPSC-derived models. Thus, to our knowledge, this is the first ever report describing the continuous effects of RA signaling on the processes of human EEC differentiation from FG.

In the present study, we demonstrated the enhancing effect of continuous activation of RA signaling on EEC induction after FG differentiation. In previous reports, the effect of RA signaling on the induction of the derivatives of FG has been controversial. We drew a comparative diagram (S-Fig. 5) showing our current protocol (a), the previous protocol for differentiation into posterior FG (PFG) (b) and ventral anterior FG (vAFG) (c) from foregut. Triso et al. [8] reported that ATRA treatment for 1 day during FG induction from DE at Day 5 in their protocol resulted in the increased expression of p63 and GATA4 at Day 9. p63 is expressed in not only dAFG but vAFG as well, which is a progenitor of lung cells [20, 31]. GATA4 is a marker of posterior FG (PFG) [8, 18, 32] but not AFG. The authors also reported that ATRA treatment for four days after Day 5 promoted posterior FG differentiation rather than AFG [8] (S-Fig 5b). Thus, which fate specification of FG into dAFG, vAFG and PFG was promoted by ATRA has been unclear. Green et al. [20] showed that six days’ treatment of FG with ATRA and CKFBE upregulated NKX2.1, a lung marker (S-Fig 5c). However, they did not assess the expression of the AFG marker SOX2, so whether ATRA treatment promotes vAFG-specific differentiation or non-specific AFG differentiation from FG has been unclear. In the present study, we demonstrated that treatment of FG with ATRA from Day 6 to Day 13 resulted in the enhanced expression of SOX2 and p63 (S-Fig. 6b), with no expression of NKX2.1 (S-Fig. 2a) or GATA4 (S-Fig. 2b). Furthermore, in our protocol, continuous treatment of FG with ATRA and CKFBE for 15–18 days enhanced neither lung markers, such as NKX2.1, SFTPB and SFTPC (Fig. 3f), nor PFG-derived stomach markers, such as TFF1, TFF2 and MUC5AC (Fig. 3g). These data indicated that ATRA promotes dAFG specification from FG. In addition, while Triso et al. [8] suggested that ATRA should be added prior to Day 6 (FG), we achieved efficient FG differentiation without ATRA by optimizing the concentration of CHIR during differentiation into DE (S-Fig. 1c and d). Differences in the identity of cells receiving ATRA among these reports might have led to these inconsistent outcomes of ATRA treatment.

No previous studies arguing the importance of retinoic acid in esophageal epithelial differentiation clarified which subtype of RA receptors played significant roles in the effect of RA. We identified for the first time RARγ as the RAR subtype whose activation promotes human EEC differentiation. This is compatible with the fact that RARγ is the predominant subtype of RAR in esophagus [18]. Regarding the differentiation of skin, which highly express RARγ (S-Fig. 3a), previous studies using mouse models reported that RA signaling via RARγ played an important role [28, 33]. In the present study, RARα and RARβ agonist treatment resulted in an increased expression of SOX2 without statistical significance, while no marked differences were noted in other esophageal markers (Fig. 4b and S-Fig. 7b). SOX2 is an early marker in the process of differentiation toward esophagus [21], and its expression is reduced in mature esophageal cells. RARα and RARβ agonist might promote the early phase of this process but not the maturation of esophageal cells. Notably, the combination of ATRA and RARγ antagonist did not increase the SOX2 expression, whereas that of RARα and RARβ agonist did. A previous study reported that ATRA binds to three types of RAR with different affinities [34]. ATRA may, therefore, have activated neither RARα nor RARβ in the current experiments, while the agonists AM580 and CD2314 activated RARα and RARβ, respectively, and enhanced the expression of SOX2.

Of note, several reports argued that RA signaling had a suppressive effect on esophageal cancer [35] as well as skin cancer [36]. In addition, the decreased expression of DSG3 [37], SPRR3 [38] and MAL [39], which were all also decreased under conditions of ATRA absence in our experiment (Fig. 3e), were reported to be related to a poor prognosis of esophageal cancer. However, no report has clarified the relationship between the prognosis and expression or mutation of RARγ in esophageal cancer. In this context, we might need to focus on other hormones affecting esophageal differentiation as well. BMP4 and EGF were reported to promote esophageal differentiation from iPSCs [7] and proliferation of esophageal keratinocytes [40], respectively, so in the final step of our differentiation protocol, we employed BMP4 and EGF as well as ATRA and omitted the reagents (CHIR + FGF10 + KGF) reported to be required for inducing differentiation into the lung [20] in order to achieve specified differentiation to the esophagus. Future studies focusing on RARγ signaling as well as these other relevant signals may provide some insight into not only esophageal differentiation but also the pathogenesis of esophageal cancer.

## Electronic supplementary material

Below is the link to the electronic supplementary material.

Supplementary file1 (TIF 7778 kb) S-Fig. 1. Gene expression of the foregut (FG) from iPSCs. (a) Expression analyses of the FG marker genes SOX2 and FOXA2 at Day 6 by semi-quantitative RT-PCR. GAPDH were used as an endogenous control. Total RNA of human normal stomach tissue was used as a positive control. (b) Representative images of immunostaining for SOX2 (green) and FOXA2 (red) at Day 6. The nuclei were stained with Hoechst (blue). Scale bars, 100 μm. (c) A schematic diagram of the experiment to examine the conditions underlying the differentiation of hiPSCs into definitive endoderm (DE) and FG. (d) qPCR of SOX2 and CDX2 in the differentiated cells treated with the indicated dose of CHIR at Day 6. GAPDH was used as an endogenous control. Data represent the mean ± SEM (n = 3). *p<0.05 from a paired t-test.

Supplementary file2 (TIF 2962 kb) S-Fig. 2. Expression of marker genes in the differentiated cells at Day 13. An expression analysis of NKX2.1 (a) and GATA4 (b) at Day 13 by semi-quantitative RT-PCR. GAPDH was used as an endogenous control. Total RNA of human normal lung tissue (a) and stomach tissue (b) were used as a positive control.

Supplementary file3 (TIF 6330 kb) S-Fig. 3. HE staining and immunohistology of the hiPSC-derived esophageal cells and fetal mouse esophagus at E17.5. HE staining and immunostaining for Claudin-4, E-cadherin and Involcrin at Day 24 in the derivatives of hiPSCs (upper panels) and fetal mouse esophagus at E17.5 (lower panels). Scale bars, 20 μm.

Supplementary file4 (TIF 3105 kb) S-Fig. 4. Esophageal differentiation with various concentrations of ATRA (a) A schematic diagram of the experiment to compare the effects of different ATRA concentrations (0, 0.1 0.5 and 1.0 μM) on EEC differentiation. (b) Expression analyses of SOX2, CK13 and FABP5 in the differentiated cells treated with 0, 0.1 0.5 and 1.0 μM of ATRA by qRT-PCR. GAPDH was used as an endogenous control. Data represent the mean ± SEM (n = 4). *p<0.05 from an ANOVA with Tukey’s test.

Supplementary file5 (TIF 5344 kb) S-Fig. 5. A schematic diagram of the effect of ATRA on organ specification from FG. (a) Our current protocol. (b) A previous protocol for differentiation into PFG. (c) A previous protocol for differentiation into vAFG.

Supplementary file6 (TIF 3826 kb) S-Fig. 6. Continuous ATRA treatment promotes differentiation into EECs. (a) A schematic diagram of the experiment to examine the effects of ATRA on the differentiation of foregut cells into dAFG cells. (b) Expression analyses of FABP5, SOX2 and p63 in differentiated cells treated with ATRA at Day 13 by qRT-PCR. GAPDH was used as an endogenous control. Data represent the mean ± SEM (n = 4). *p<0.05 from a paired t-test. (c) A schematic diagram of the experiment to address the effect of ATRA on the differentiation of dAFG into EEC ((i) no addition (ii) from Day 6 to Day 13, (iii) from Day 17 to Day 24 (iv) from Day 6 to Day 24). (d) Expression analyses of CK13 and FABP5 in the differentiated cells treated with ATRA at Day 24 by qRT-PCR. GAPDH was used as an endogenous control. Data represent the mean ± SEM (n = 5). *p<0.05 from an ANOVA with Tukey’s test.

Supplementary file7 (TIF 5100 kb) S-Fig. 7. The expressions of RARα, RARβ and RARγ in human tissues and effect of RARγ antagonist on EEC differentiation. (a) The bar graphs show the average FPKM levels of the RAR subtypes (RARα, RARβ and RARγ) in various human tissues. (b and c) Expression analyses of FABP5, SOX2, CK13, PAX9 and S100A14 in the differentiated cells treated with CD2314 (RARβ agonist) and MM11253 (RARγ antagonist) by qRT-PCR. GAPDH was used as an endogenous control. Data represent the mean ± SEM (n = 5). *p<0.05 from an ANOVA with Tukey’s test.

Supplementary file8 (TIF 3924 kb) S-Fig. 8. Expression of stomach and neuronal cell marker genes in the differentiated cells. (a) An expression analysis of the stomach marker genes SOX2, TFF1, TFF2 and MUC5AC at Day 21 by semi-quantitative RT-PCR. GAPDH was used as an endogenous control. Total RNA of human normal stomach tissue was used as a positive control. (b) An expression analysis of the neuronal cell marker genes SOX2, SOX1 and PAX6 at Day 21 by semi-quantitative RT-PCR. GAPDH was used as an endogenous control. Total RNA of human normal brain tissue was used as a positive control.

Supplementary file9 (TIF 4276 kb) S-Fig. 9. HE staining and immunohistology of the hiPSC-derived esophageal cells treated with an RARγ-specific agonist instead of ATRA. HE staining and immunostaining for Claudin-4, E-cadherin and Involcrin at Day 24.

## Abbreviations

iPSCs: Induced pluripotent stem cells
RA: Retinoic acid
EEC: Esophageal epithelial cell
ATRA: All-trans retinoic acid
RAR: Retinoic acid receptor
RT-PCR: Reverse transcription polymerase chain reaction
PBMCs: Peripheral blood mononuclear cells
DE: Definitive endoderm
FG: Foregut
dAFG: Dorsal anterior foregut
ALI: Air–liquid interface
TPM: Transcripts per million
FPKM: Fragments per kilobase of exon per million mapped fragments
PFG: Posterior FG
vAFG: Ventral anterior foregut
