# Supplementary figures and images for: Retinoic acid receptor γ activation promotes differentiation of human induced pluripotent stem cells into esophageal epithelium
Source: J Gastroenterol. 2020 Jun 16;55(8):763–74. doi: 10.1007/s00535-020-01695-7 (PMC7376085; doi:10.1007/s00535-020-01695-7)

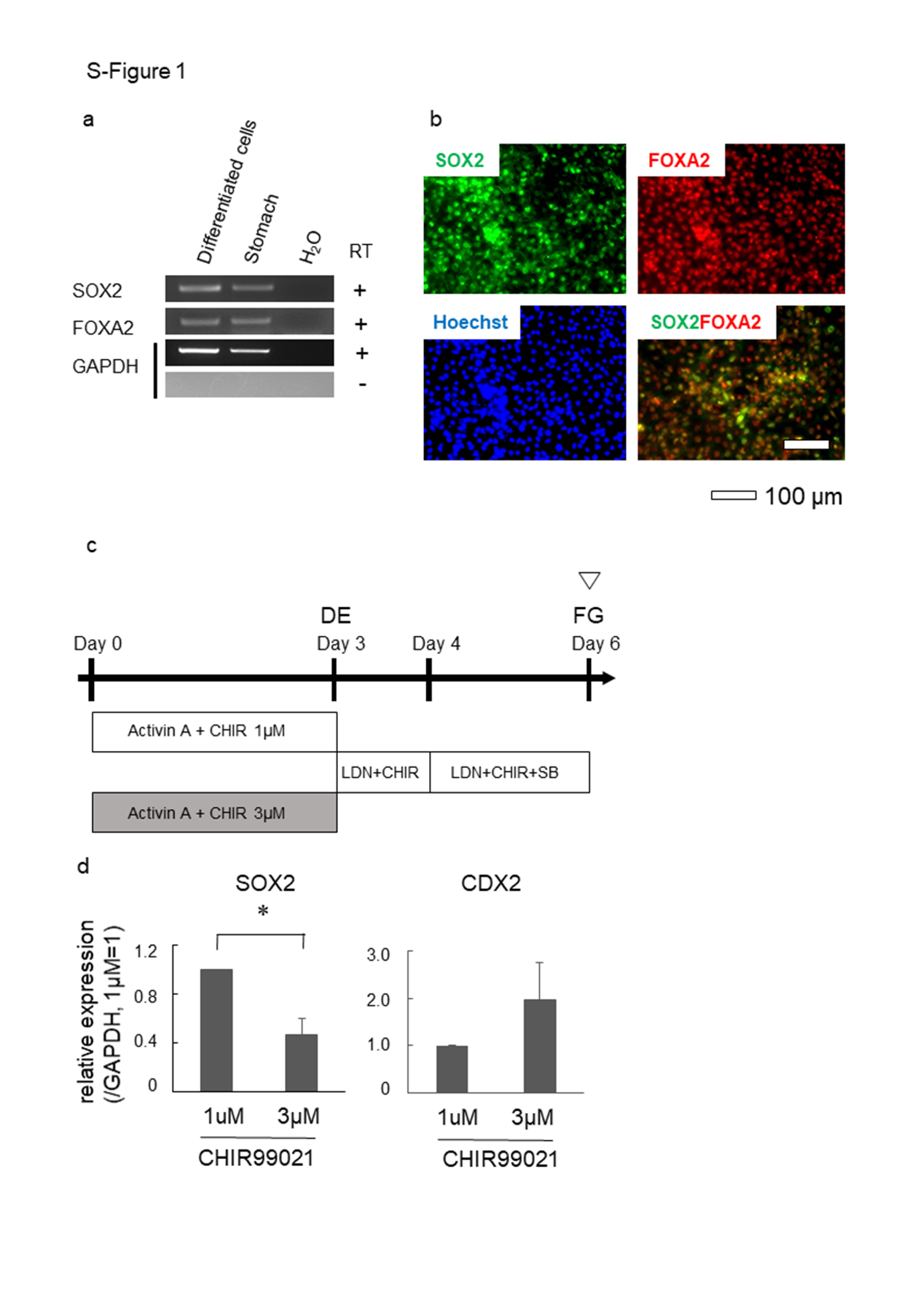

Supplement: Supplementary file 1 — Supplementary file1 (TIF 7778 kb) S-Fig. 1. Gene expression of the foregut (FG) from iPSCs. (a) Expression analyses of the FG marker genes SOX2 and FOXA2 at Day 6 by semi-quantitative RT-PCR. GAPDH were used as an endogenous control. Total RNA of human normal stomach tissue was used as a positive control. (b) Representative images of immunostaining for SOX2 (green) and FOXA2 (red) at Day 6. The nuclei were stained with Hoechst (blue). Scale bars, 100 μm. (c) A schematic diagram of the experiment to examine the conditions underlying the differentiation of hiPSCs into definitive endoderm (DE) and FG. (d) qPCR of SOX2 and CDX2 in the differentiated cells treated with the indicated dose of CHIR at Day 6. GAPDH was used as an endogenous control. Data represent the mean ± SEM (n = 3). *p<0.05 from a paired t-test. [file 535_2020_1695_MOESM1_ESM.tif]

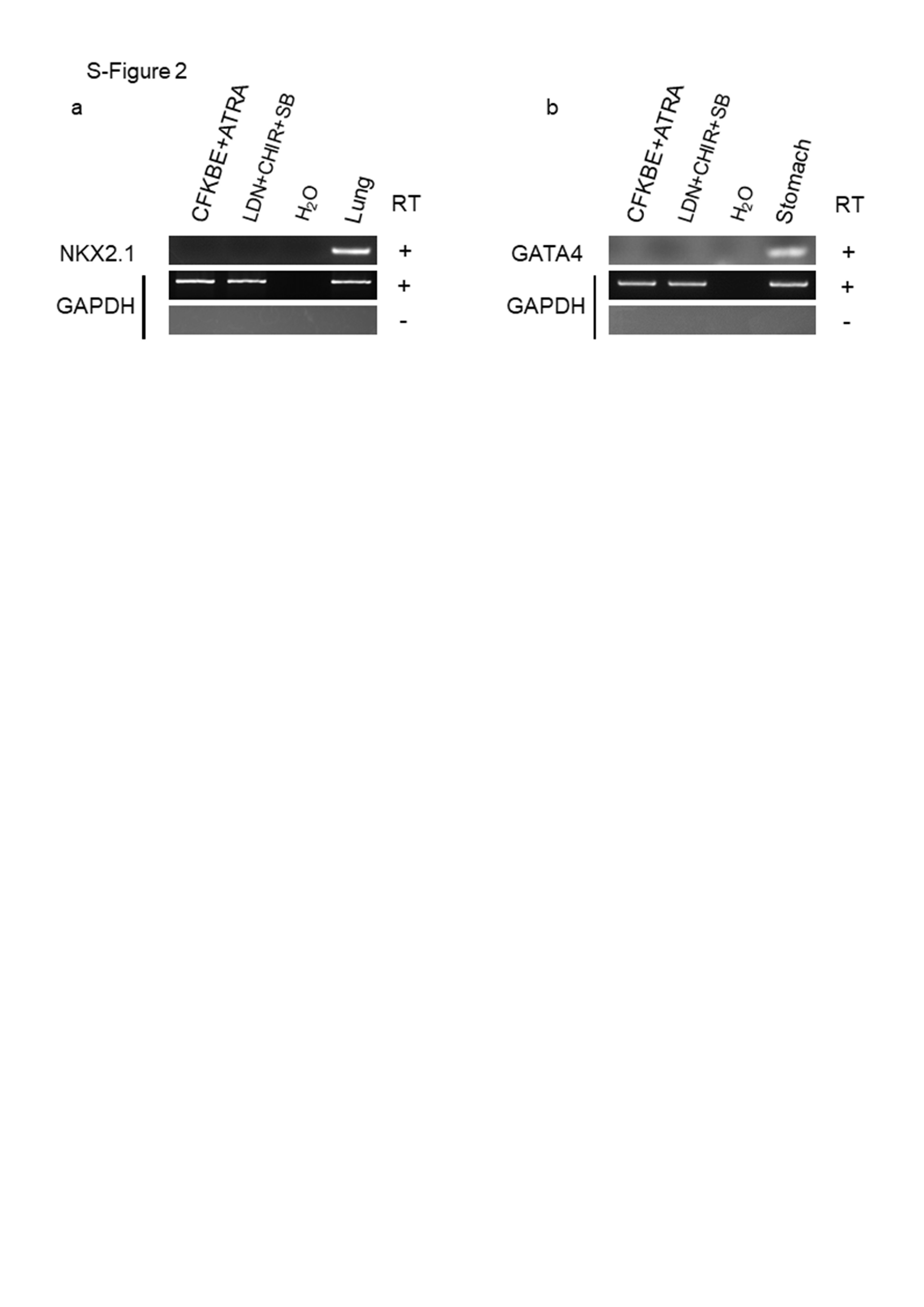

Supplement: Supplementary file 2 — Supplementary file2 (TIF 2962 kb) S-Fig. 2. Expression of marker genes in the differentiated cells at Day 13. An expression analysis of NKX2.1 (a) and GATA4 (b) at Day 13 by semi-quantitative RT-PCR. GAPDH was used as an endogenous control. Total RNA of human normal lung tissue (a) and stomach tissue (b) were used as a positive control. [file 535_2020_1695_MOESM2_ESM.tif]

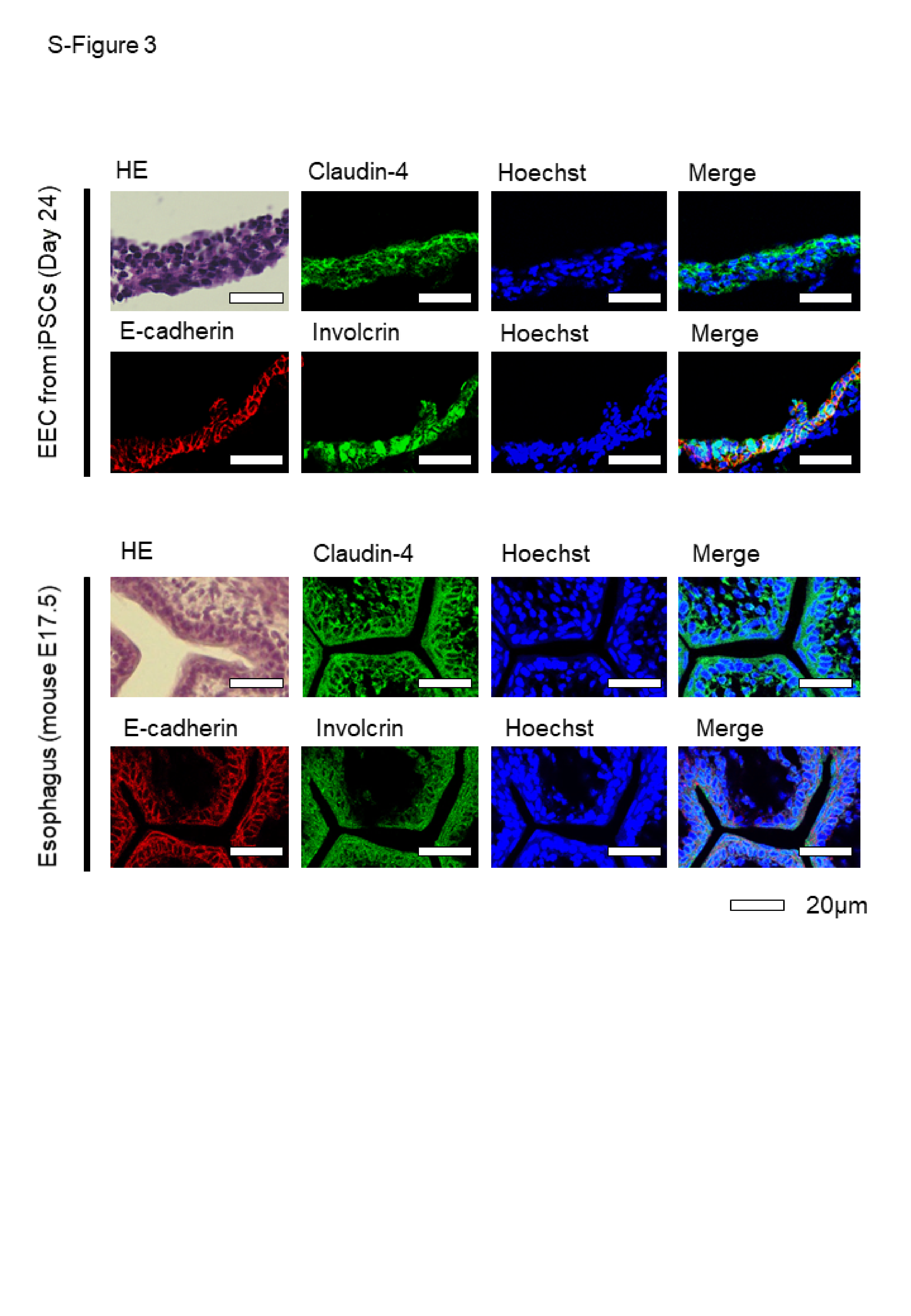

Supplement: Supplementary file 3 — Supplementary file3 (TIF 6330 kb) S-Fig. 3. HE staining and immunohistology of the hiPSC-derived esophageal cells and fetal mouse esophagus at E17.5. HE staining and immunostaining for Claudin-4, E-cadherin and Involcrin at Day 24 in the derivatives of hiPSCs (upper panels) and fetal mouse esophagus at E17.5 (lower panels). Scale bars, 20 μm. [file 535_2020_1695_MOESM3_ESM.tif]

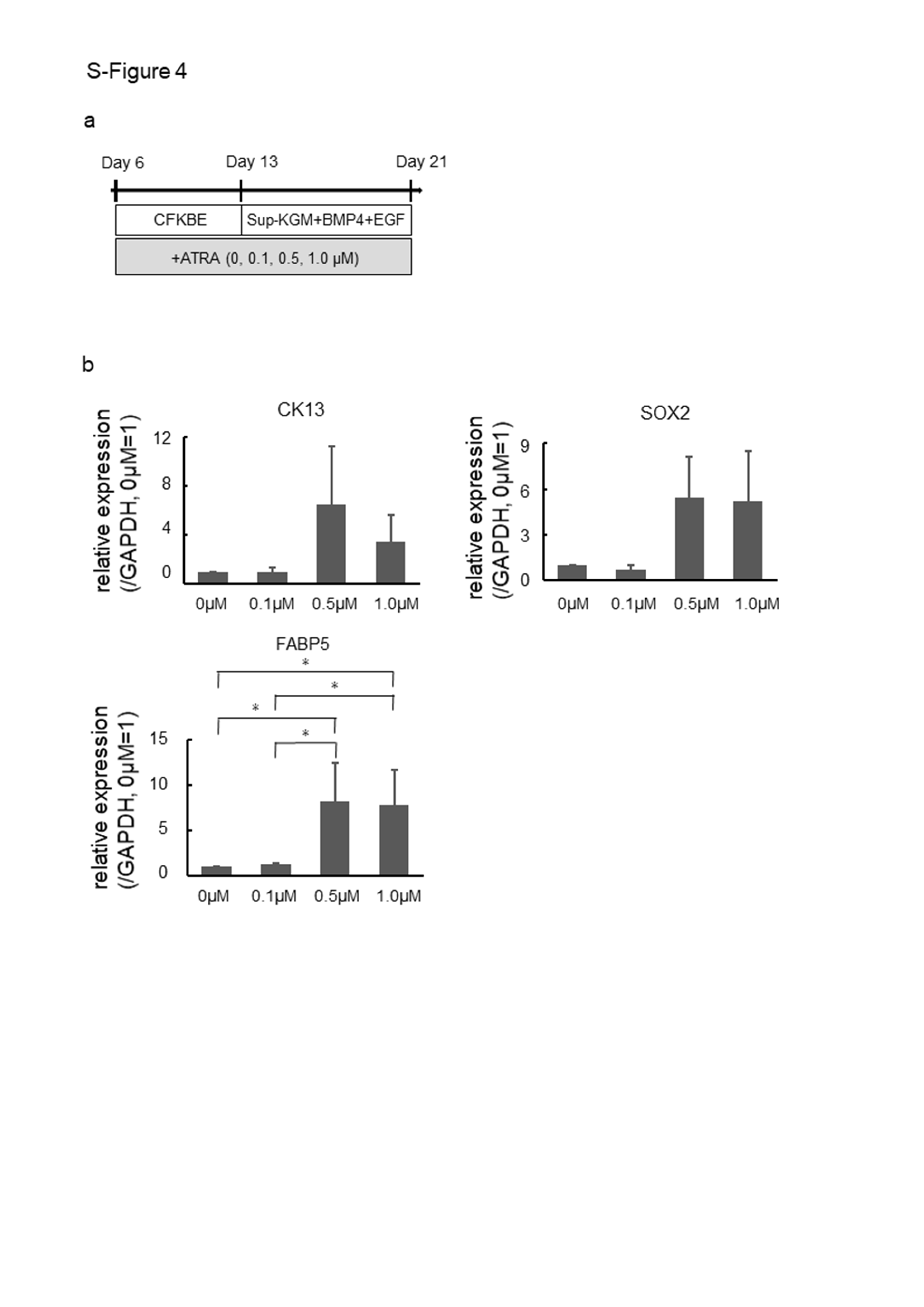

Supplement: Supplementary file 4 — Supplementary file4 (TIF 3105 kb) S-Fig. 4. Esophageal differentiation with various concentrations of ATRA (a) A schematic diagram of the experiment to compare the effects of different ATRA concentrations (0, 0.1 0.5 and 1.0 μM) on EEC differentiation. (b) Expression analyses of SOX2, CK13 and FABP5 in the differentiated cells treated with 0, 0.1 0.5 and 1.0 μM of ATRA by qRT-PCR. GAPDH was used as an endogenous control. Data represent the mean ± SEM (n = 4). *p<0.05 from an ANOVA with Tukey’s test. [file 535_2020_1695_MOESM4_ESM.tif]

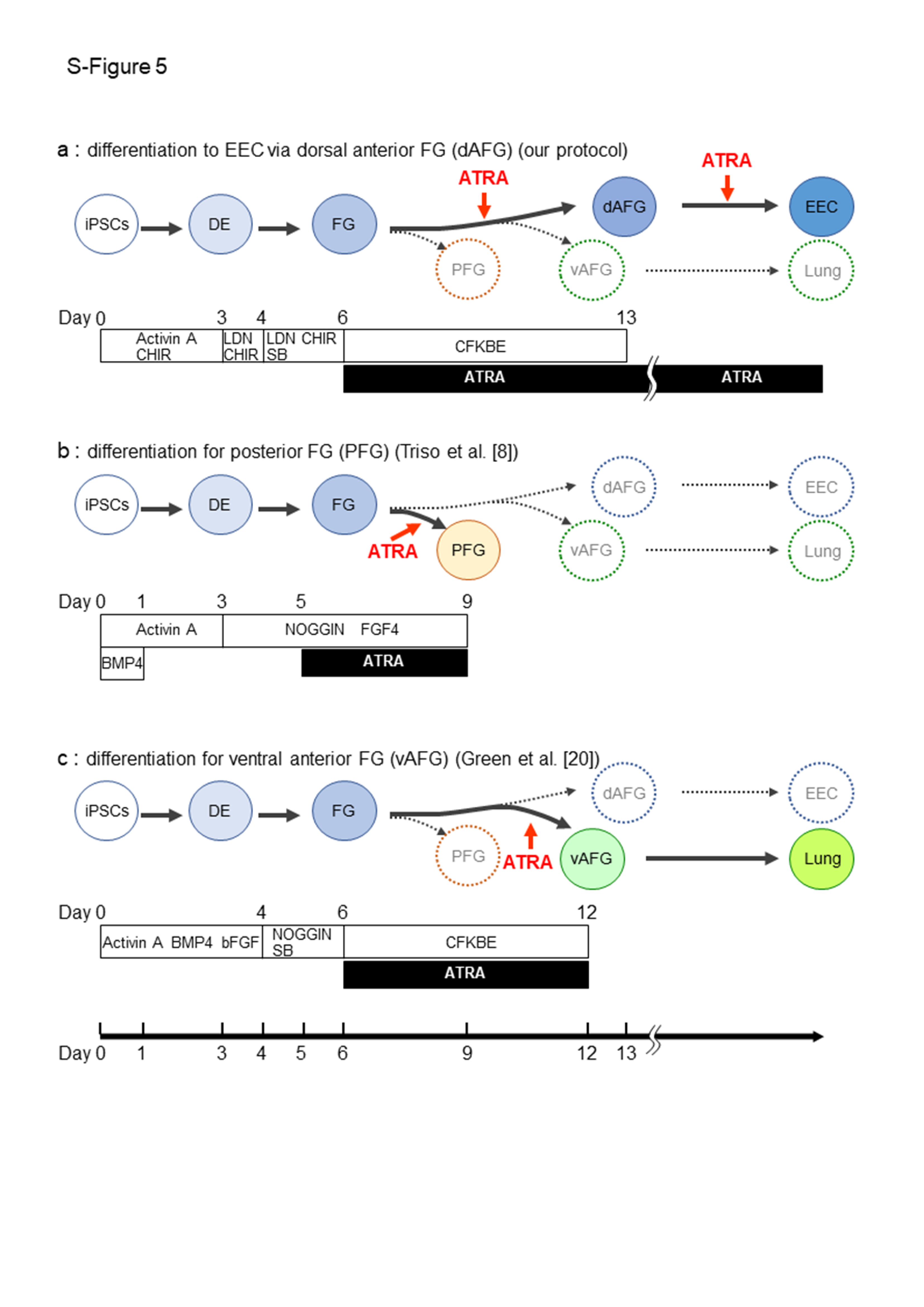

Supplement: Supplementary file 5 — Supplementary file5 (TIF 5344 kb) S-Fig. 5. A schematic diagram of the effect of ATRA on organ specification from FG. (a) Our current protocol. (b) A previous protocol for differentiation into PFG. (c) A previous protocol for differentiation into vAFG. [file 535_2020_1695_MOESM5_ESM.tif]

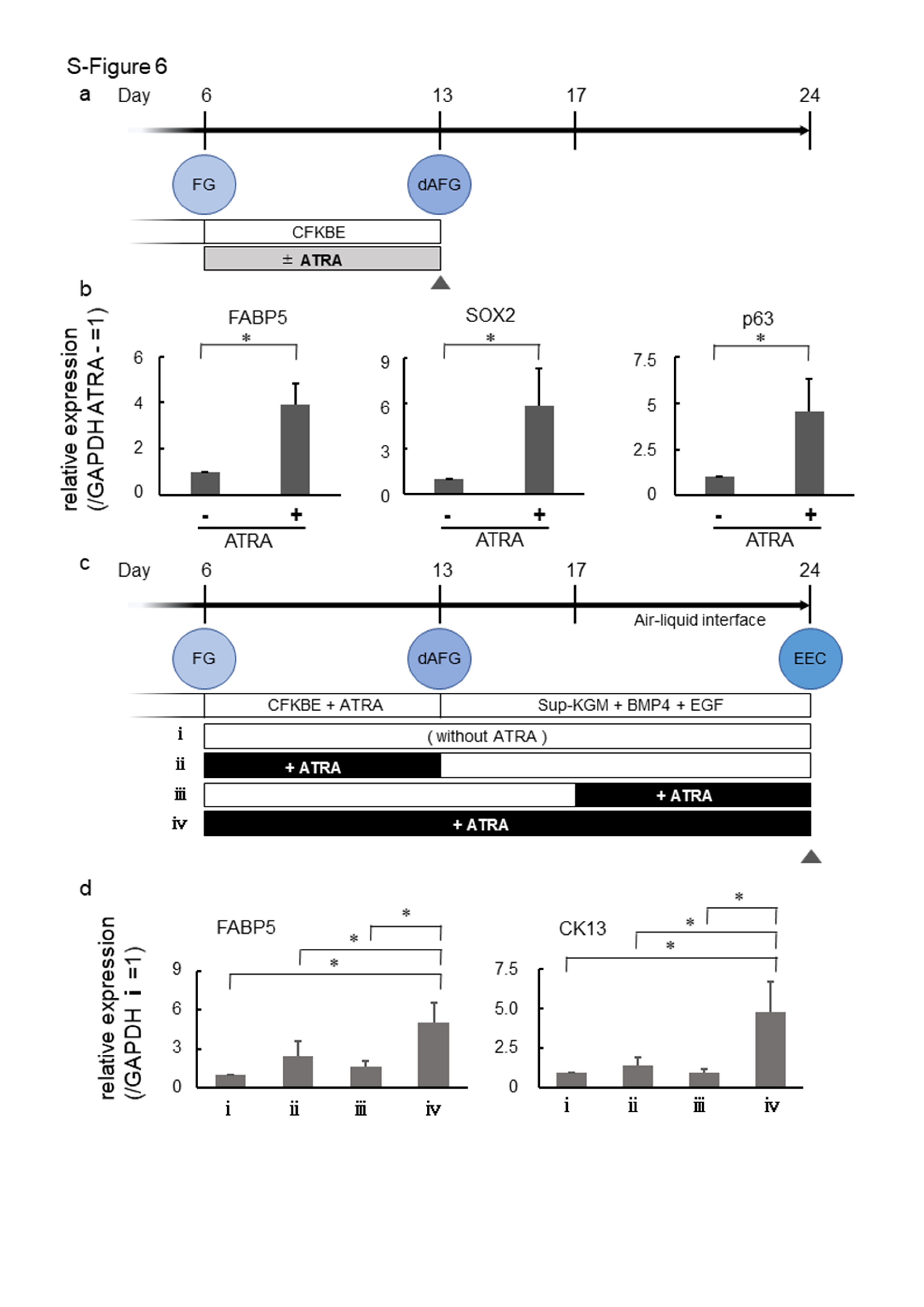

Supplement: Supplementary file 6 — Supplementary file6 (TIF 3826 kb) S-Fig. 6. Continuous ATRA treatment promotes differentiation into EECs. (a) A schematic diagram of the experiment to examine the effects of ATRA on the differentiation of foregut cells into dAFG cells. (b) Expression analyses of FABP5, SOX2 and p63 in differentiated cells treated with ATRA at Day 13 by qRT-PCR. GAPDH was used as an endogenous control. Data represent the mean ± SEM (n = 4). *p<0.05 from a paired t-test. (c) A schematic diagram of the experiment to address the effect of ATRA on the differentiation of dAFG into EEC ((i) no addition (ii) from Day 6 to Day 13, (iii) from Day 17 to Day 24 (iv) from Day 6 to Day 24). (d) Expression analyses of CK13 and FABP5 in the differentiated cells treated with ATRA at Day 24 by qRT-PCR. GAPDH was used as an endogenous control. Data represent the mean ± SEM (n = 5). *p<0.05 from an ANOVA with Tukey’s test. [file 535_2020_1695_MOESM6_ESM.tif]

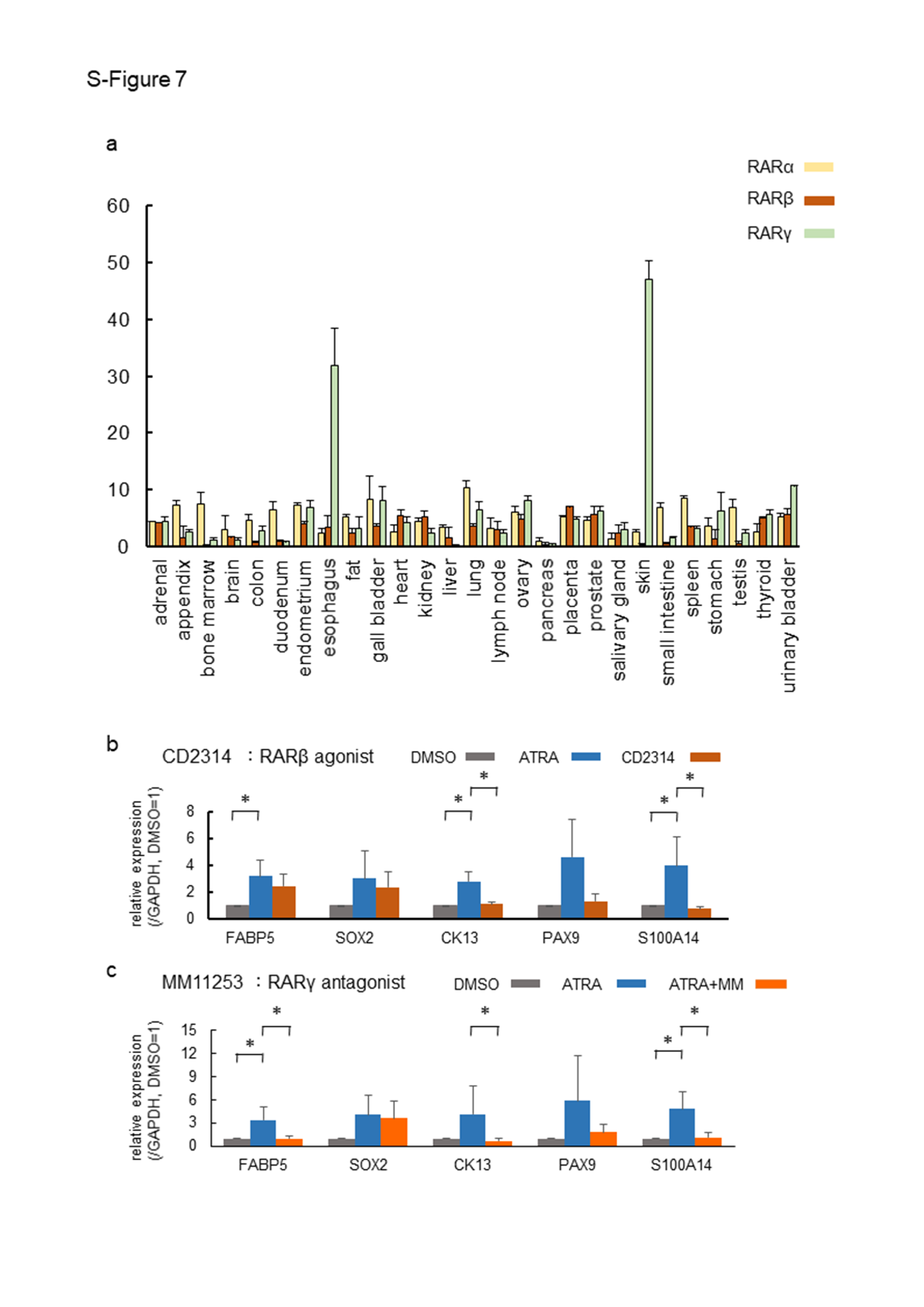

Supplement: Supplementary file 7 — Supplementary file7 (TIF 5100 kb) S-Fig. 7. The expressions of RARα, RARβ and RARγ in human tissues and effect of RARγ antagonist on EEC differentiation. (a) The bar graphs show the average FPKM levels of the RAR subtypes (RARα, RARβ and RARγ) in various human tissues. (b and c) Expression analyses of FABP5, SOX2, CK13, PAX9 and S100A14 in the differentiated cells treated with CD2314 (RARβ agonist) and MM11253 (RARγ antagonist) by qRT-PCR. GAPDH was used as an endogenous control. Data represent the mean ± SEM (n = 5). *p<0.05 from an ANOVA with Tukey’s test. [file 535_2020_1695_MOESM7_ESM.tif]

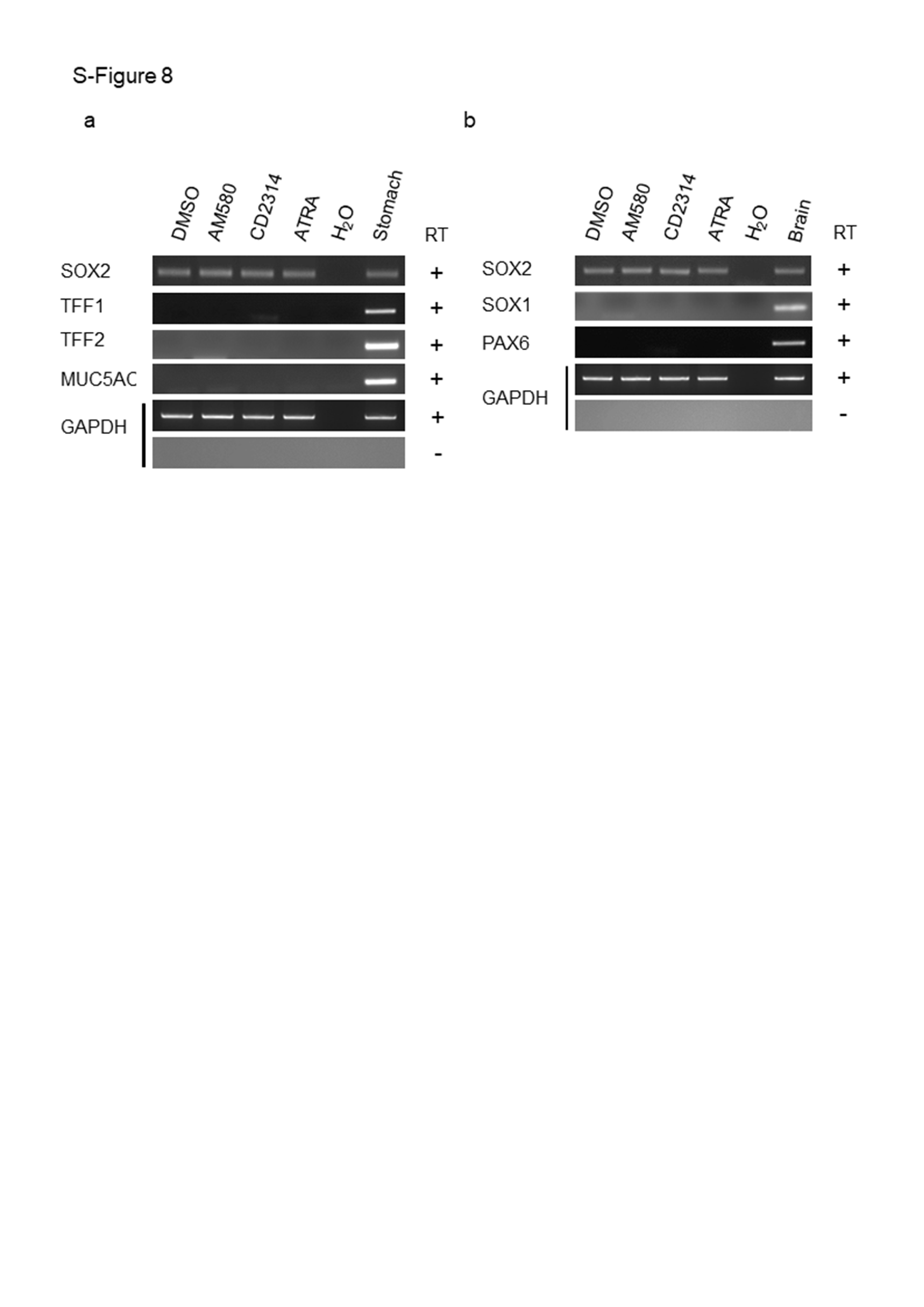

Supplement: Supplementary file 8 — Supplementary file8 (TIF 3924 kb) S-Fig. 8. Expression of stomach and neuronal cell marker genes in the differentiated cells. (a) An expression analysis of the stomach marker genes SOX2, TFF1, TFF2 and MUC5AC at Day 21 by semi-quantitative RT-PCR. GAPDH was used as an endogenous control. Total RNA of human normal stomach tissue was used as a positive control. (b) An expression analysis of the neuronal cell marker genes SOX2, SOX1 and PAX6 at Day 21 by semi-quantitative RT-PCR. GAPDH was used as an endogenous control. Total RNA of human normal brain tissue was used as a positive control. [file 535_2020_1695_MOESM8_ESM.tif]

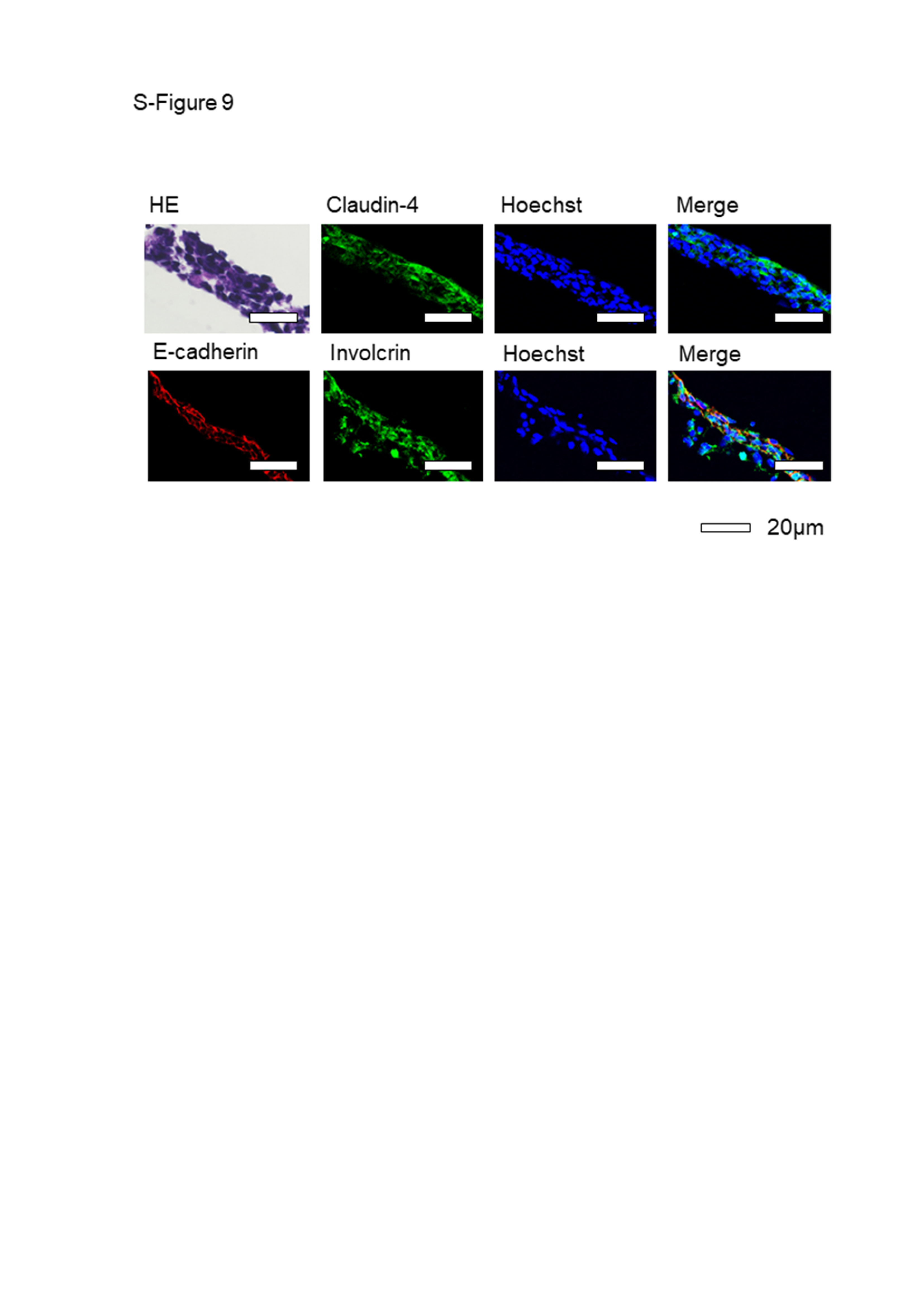

Supplement: Supplementary file 9 — Supplementary file9 (TIF 4276 kb) S-Fig. 9. HE staining and immunohistology of the hiPSC-derived esophageal cells treated with an RARγ-specific agonist instead of ATRA. HE staining and immunostaining for Claudin-4, E-cadherin and Involcrin at Day 24. [file 535_2020_1695_MOESM9_ESM.tif]
